# An open-source research tool to study triaxial inertial sensors for monitoring selected behaviors in sheep

**DOI:** 10.1093/tas/txaa188

**Published:** 2020-10-09

**Authors:** Barbara R dos Reis, Daniel R Fuka, Zachary M Easton, Robin R White

**Affiliations:** 1 Department of Animal and Poultry Sciences, Virginia Tech, Blacksburg, VA; 2 Department of Biological Systems Engineering, Virginia Tech, Blacksburg, VA

**Keywords:** precision technology, sensor, validation

## Abstract

The use of automated systems for monitoring animal behavior provides information on individual animal behavior and can be used to enhance animal productivity. However, the advancement of this industry is hampered by technology costs, challenges with power supplies, limited data accessibility, and inconsistent testing approaches for confirming the detection of livestock behaviors. Development of open-source research tools similar to commercially available wearable technologies may contribute to the development of more-efficient and affordable technologies. The objective of this study was to demonstrate an open-source, microprocessor-based sensor designed to monitor and enable differentiation among selected behaviors of adult wethers. The sensor was comprised of an inexpensive espressif ESP-32-WROOM-32 microprocessor with Bluetooth communication, a generic MPU92/50 motion sensor that contains a three-axis accelerometer, three-axis magnetometer, a three-axis gyroscope, and a 5-V rechargeable lithium-ion battery. The open-source Arduino IDE software was used to program the microprocessor and to adjust the frequency of sampling, the data packet to send, and the operating conditions. For demonstration purposes, sensors were placed on six housed sheep for three 1-h increments with concurrent visual behavioral observation. Sensor readings (x-, y-, and z-axis) were summarized (mean and SD) within a minute and compared to animal behavior observations (also on a by-minute basis) using a linear mixed-effect model with animal as a random effect and behavioral classifier as a fixed effect. This analysis demonstrated the basic utility of the sensor to differentiate among animal behaviors based on sensed data (*P* < 0.001). Although substantial additional work is needed for algorithm development, power source testing, and network optimization, this open-source platform appears to be a promising strategy to research wearable sensors in a generalizable manner.

## INTRODUCTION

In the past years, the use of precision technologies in livestock systems has been increasing as an attempt to enhance animal productivity. Understanding and observing animal behaviors can be an indicator of animal growth, health status (González et al., 2008; Neethirajan, 2017), welfare (Rushen et al., 2012), reproduction (Valenza et al., 2012; Andersson et al., 2016), and their interaction with the environment (Handcock et al., 2009). Therefore, the recognition of individual animal behaviors is extremely important to facilitate management and urgent interventions. In real-world production situations, observing animals continuously is impracticable, if not impossible, in addition to being time and labor intensive. Moreover, direct observations of animals have the limitation of providing behavioral estimations only during the observation time, meaning that the behavioral states associated with health, welfare, and other conditions may not be noticed during the inspection (Barwick et al., 2018). In this context, wearable sensor technologies show promise over traditional animal behavior measurements due to the possibility of managing individual animals without any physical involvement. If low cost and scalable, these technologies can also be easily upscaled to the monitoring of large herds.

Wearable sensor network systems consist of one or multiple nodes equipped with one or more sensors, a power supply, a processor, a transceiver (Bluetooth, radio, and Wi-fi), and a memory (Yick et al., 2008). The nodes of the sensors are able to measure and collect information of interest from sensors within the environment and transmit the data to the user (Yick et al., 2008). Several wearable commercial sensors are available (Cow Intelligence, 2020; CowManager, 2020; HealthyCow24, 2020; Herd Intelligence, 2020; MooMonitor+, 2020) that classify animal behaviors and generate warnings in case of necessary management intervention. Although these technologies reduce time and labor costs, the price associated with acquiring these devices is high. For example, commercial GPS collar systems cost $1,500 to 2,000 per animal (Anderson et al., 2013). Another important challenge for the advancement of these sensor networks is the accuracy of their analytics. More accurate analytics can be achieved with more frequent sampling and data transmission; however, sampling and edge processing frequently can be power intensive. As such, there is a need to develop an approach to maintain adequate levels of accuracy and reduce the power requirement of sampling and data analysis (Radeski and Ilieski, 2017). A final challenge associated with commercial wearable sensors is the data interpretation and ownership. Systems tend to either provide raw data, which is challenging for interpretation in commercial uses, or “cleaned” data only, which often relies on proprietary classification algorithms and data cleaning approaches, making research with a sensor system applicable only to that system and to that iteration of algorithm. To make better use of data collected, there is a need to enhance the flexibility of data reporting and storage options for research activities. As such, it is likely that an open-source research platform for wearable sensor networks may be a useful addition to the suite of technology options available, making research more applicable across commercial platforms and enhancing opportunities to collectively advance this industry.

In this technical note, we present the design of an open-source sensor system to investigate and record animal behavior using triaxial inertial measurement units. The proposed prototype sensor is low cost and allows for flexible, developmental research on approaches to enhancing power supply, data transmission, and expansion of sensing capacity. Furthermore, the use of generic parts allows us to begin developing data libraries for the monitoring of animal behaviors, which can be leveraged across wearable sensor platforms for improved algorithm development and testing. The aim of the present study was to compare the readings of this generic low-cost, low-power Bluetooth sensor equipped with 9-df inertial motion sensing capacity when adult wethers were lying, standing, eating, drinking, and ruminating. Although several studies have demonstrated that commercial inertial sensors are capable of differentiating among these behaviors, this preliminary demonstration of our research sensor system is essential to demonstrate viability and comparability to existing products. After this preliminary investigation, further work can be conducted to enhance power use efficiency, expand sensing capacity, build data libraries, and conduct other essential precompetitive research objectives. Based on the success of commercial sensors at differentiating behaviors, we hypothesized that an open-source sensor would generate readings that differed significantly between ruminating, drinking, lying, standing, and eating.

## MATERIAL AND METHODS

### System General Description

**Figure 1.**
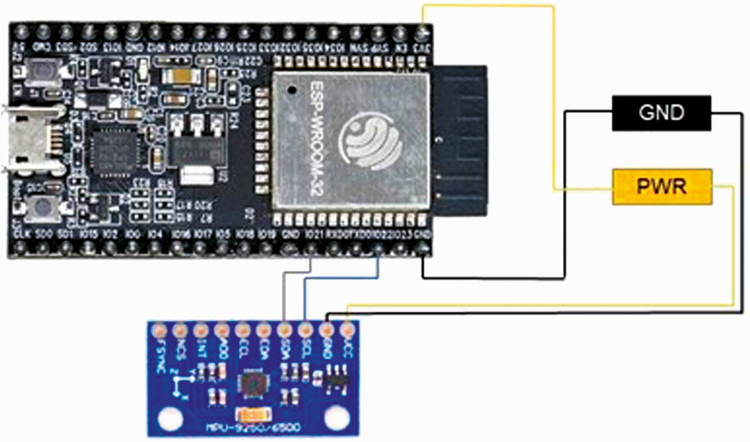
Diagram for connections among the microprocessor and the motion sensor. The wires are represented by different colors. The wires to and from the power bus and the wires to and from the ground bus are represented in yellow and black, respectively.

The wearable sensor (Fig. 1) comprised of an Espressif ESP-32-WROOM-32 microprocessor with Wi-Fi and Bluetooth communication, a generic MPU92/50 motion sensor which contains a three-axis accelerometer, three-axis magnetometer, and a three-axis gyroscope, and a 5-V rechargeable lithium-ion battery. All connections in Fig. 1 were made using 1.0-mm solder (63% tin, 27% lead, and 1.8% flux rosin core); 22 gauge, PVC insulated solid wire; and a Weller WLC100 40-W soldering iron. The microprocessor was designed to be programmed flexibly by researchers using the open-source Arduino IDE software to adjust the sensor operations according to the experimental conditions, including the frequency of sampling, the battery life draw, and handling of data between sampling events.

### Programming the Wearable Sensor

**Figure 2.**
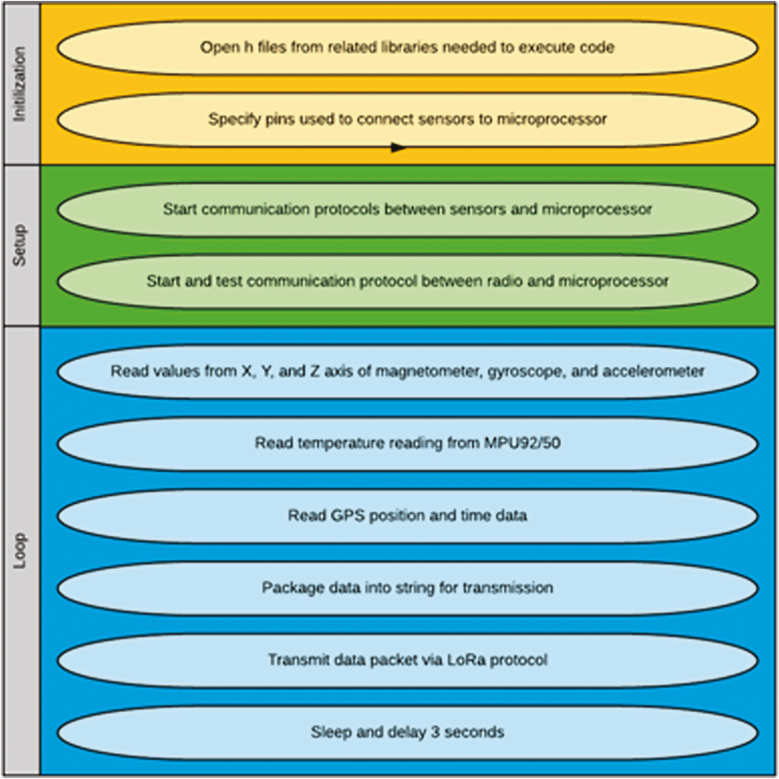
Flowthrough of process used to program the microprocessor. Each oval summarized the individual executable actions and the code sections are highlighted by different colors.

The open-source Arduino IDE software (https://www.arduino.cc/en/Main/Software) was used to program the sensors via the universal serial bus port. Briefly, the code was divided into three sections: code designed to be performed upon initialization; setup code; and looping code. Figure 2 graphically represents the processes incorporated in the wearable sensor code. The maximum string length for strings sent via Bluetooth was established to 250 characters and the serial baud was set to 115,200 bits per second. The serial communication between the microcontroller, the Bluetooth transmission, and the MPU92/50 were initialized in the setup portion of the code. The loop code was programmed to iterate repeatedly so that the data from the sensor was expected to be sent to the paired Bluetooth receiver 10 times per second. The data sent through the serial Bluetooth connection included readings obtained from the x-, y-, and z-axes of the accelerometer, magnetometer, and gyroscope on board the MPU92/50. The open-source Arduino library MPU9250_asukiaaa was used to convert voltage readings from the MPU92/50 into interpretable inertial sensor readings. To retrieve and send data, the SoftwareSerial and SPI libraries of the Arduino IDE were used.

### Experimental Site and Animals

**Figure 3.**
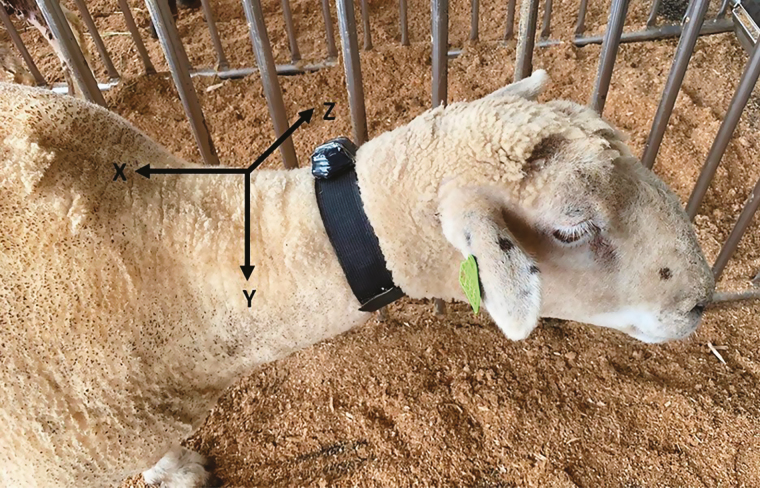
Experimental animal displaying the location and orientation of the prototype sensor evaluated in the study.

All animal experimental procedures conducted in this study were approved by the Virginia Polytechnic Institute and State University Animal Care and Use Committee (protocol #19–159). The current study was conducted between May and June of 2020 at the Smithfield Farm, Virginia Polytechnic Institute and State University, Blacksburg, VA. The prototype sensor discussed in the previous sections was deployed on a neck collar (Fig. 3) and used to monitor the behavior of six housed adult crossbred Suffolk × Dorset wethers, with an average weight of 70 ± 5 kg (mean ± SD). The animals were housed individually (10 × 3 ft cages) and could easily move around the cage. Grass hay (91.6% dry matter) and clean water were available ad libitum.

### Sensor Data Collection and Direct Behavior Observation

Each animal was equipped with a prototype sensor for three 1-h sampling periods. Each sensor was programmed with a unique ID and connected to a local computer via Bluetooth. The Free Serial Monitor (https://www.com-port-monitoring.com/downloads.html) software was used to log data sent over the Bluetooth connection to a local text file on the computer. Although not used in this study, the system can also be used with a Serial Port Monitor on a cell phone if a computer in the research area is not available or not feasible. Direct behavioral observations (DO) were coded by a single observer for all sampling periods. The observer classified behaviors based on the way the animal spent its time during the majority of each minute. Behaviors evaluated included: 1) standing: static standing with no jaw movements; 2) laying: laying down in a resting position with no other movements; 3) rumination: when the animal was laying or standing while regurgitation, chewing, and swallowing of ingested feed bolus; 4) eating: feed intake from the feed bucket and chewing and swallowing of feed; and 5) drinking: swallowing the water from the water bucket. The minute-by-minute DO were recorded manually by the observer in a spreadsheet and cross-referenced to the sensor data based on the recording time. The sensor was aligned on the collar such that the x-axis was aligned along the cranial/caudal axis; the y-axis was aligned left to right; and the z-axis was aligned up/down.

### Statistical Analysis

Statistical analysis was performed using R Statistical Software v3.6.1 (R Core Team, 2019) and the lme4 package (Bates et al., 2015) was used to conduct the model derivation. Relationships were analyzed using a linear mixed-effects model with animal as a random effect and behavioral classifier as a fixed effect. The three-axis readings of the inertial sensors (accelerometer, magnetometer, and gyroscope) were used as the response variables. Analysis of variance was performed on the model and least-squares means were estimated from the emmeans package (Lenth et al., 2018). Significance was declared at *P* < 0.05 and a tendency considered when 0.05 ≤ *P* ≤ 0.10.

## RESULTS AND DISCUSSION

### System Technical Evaluation

The primary purpose of this system is to provide a flexible, open-source platform for investigating technical and data-based advancements for wearable animal sensors. The system takes about 10 min for a moderately experienced technician to construct if all parts and supplies are on hand. Training needed to progress from a complete novice to being capable of independently constructing a sensor took undergraduate student volunteers typically less than an hour. As such, the system has high promise as a platform that can be constructed and augmented by researchers with ease. The current commodity costs for the batteries ($12), microprocessor ($11), and nine-axis inertial sensor ($8) also mean that this system is low cost to construct.

Networking flexibility and battery life are major challenges that limit the utility of commercial wearable sensors. The networking technologies and expected battery life of this research platform are not expected to compete with commercial wearable sensors; however, the flexibility of the platform allows for experimentation to evaluate strategies to optimize the power efficiency of the sensor, to alter power sources, or to change networking technologies and data transmission rates. Indeed, the flexibility of the Arduino-based microprocessor platform enables the testing of multiple data transmission modes (Bluetooth, Wi-Fi, LoRa, etc.) independently or simultaneously. Alterations in battery size and type are easily accomplished, as are additions of solar power support or other energy harvesting technologies.

### Characterization of Animal Behaviors

In addition to the requirement of the system being easy to assemble, flexible, and low cost, a wearable sensor system to facilitate research advancements must show initial promise in differentiating among animal behaviors. Main daily activities of ruminants include eating, ruminating, and resting; therefore, these behaviors were the target of our behavioral observation using the sensor data. These behaviors are particularly important because changes in their duration or frequency can be a potential way to monitor welfare and health status of ruminants (Weary et al., 2009; Medrano-Galarza et al., 2012; Blackie and Maclaurin, 2019). The least-square means for sensor data for the recorded behaviors are shown in Table 1. The significant differences among individual axis readings for each behavior highlight strategies to leverage the sensor data for behavioral classification. As we develop more sophisticated algorithms for the characterization of animal behaviors, these can be uploaded to the sensor to enable real-time behavioral classification and associated research on the precision and accuracy of these approaches.

**Table 1.** Least square means (±SE) for the recorded behaviors obtained from the axis x–y–z from the accelerometer, magnetometer, and gyroscope from wethers during the experimental period

	Standing		Laying		Eating		Ruminating		Drinking		
Behavior classifier	Lsmean	95% CI	Lsmean	95% CI	Lsmean	95% CI	Lsmean	95% CI	Lsmean	95% CI	*P*-value
Variable											
Mean											
Accelerometer x-axis	−0.09	−5.37, 5.16	−5.46	−11.21, 0.273	−1.85	−7.15, 3.440	−2.58	−7.83, 2.66	−1.91	−7.09, 3.25	<0.001
Accelerometer y-axis	−1.84	−4.87, 1.17	4.08	−0.58, 8.77	−1.57	−4.63, 1.49	2.36	−0.64, 5.38	−0.61	−3.78, 2.55	<0.001
Accelerometer z-axis	5.09	2.92, 7.25	6.27	3.51, 9.02	5.02	2.83, 7.20	5.40	3.24, 7.55	5.4	3.24, 7.56	0.56
Magnetometer x-axis	63.4	23.8, 96.4	67.0	31.6, 102.5	60.1	23.8, 96.4	67.1	30.8, 103.3	63.6	27.6, 99.6	<0.001
Magnetometer y-axis	22.7	7.69, 36.8	3.58	−14.77, 21.8	15.49	0.78, 30.2	9.27	−5.25, 23.80	12.34	−2.18, 26.90	<0.001
Magnetometer z-axis	−7.78	−25.2, 9.67	−11.76	−30.8, 7.25	−12.86	−30.4, 4.69	−10.10	−27.5, 7.31	−10.09	−27.2, 7.07	<0.01
Gyroscope x-axis	−0.1	−0.19, −0.01	−0.16	−0.26, −0.06	−0.12	−0.22, −0.03	−0.12	−0.21, −0.03	−0.11	−0.20, −0.02	0.01
Gyroscope y-axis	0.05	−0.26, 0.37	0.06	−0.25, 0.38	0.06	−0.26, 0.38	0.05	−0.26, 0.38	0.04	−0.27, 0.36	0.103
Gyroscope z-axis	−0.13	−0.37, 0.10	−0.23	−0.46, 0.001	−0.18	−0.42, 0.05	−0.17	−0.41, 0.06	−0.17	−0.40, 0.06	<0.001
SD											
Accelerometer x-axis	1.81	1.39, 2.92	0.75	−0.84, 2.36	2.29	1.94, 2.64	1.10	0.64, 1.57	2.15	1.39, 2.92	<0.001
Accelerometer y-axis	3.37	2.39, 4.35	1.98	0.206, 3.76	3.82	2.82, 4.81	2.43	1.45, 3.41	3.62	2.53, 4.71	<0.001
Accelerometer z-axis	1.85	0.72, 2.98	0.94	−0.49, 2.40	2.11	0.97, 3.25	1.21	0.08, 2.34	2.11	0.98, 3.24	<0.001
Magnetometer x-axis	8.56	6.35, 10.77	4.85	1.38, 8.32	8.38	6.14, 10.62	6.47	4.26, 8.67	7.89	5.56, 10.21	<0.001
Magnetometer y-axis	13.85	10.18, 17.5	8.88	1.25, 16.5	15.98	12.27, 19.7	10.74	7.06, 14.4	13.8	9.44, 18.2	<0.001
Magnetometer z-axis	9.50	4.19, 14.8	4.80	−1.07, 10.70	8.64	3.30, 14.0	7.62	2.33, 12.9	7.93	2.72, 13.1	<0.001
Gyroscope x-axis	0.4	0.29, 0.50	0.16	0.01, 0.31	0.46	0.36, 0.57	0.26	0.16, 0.37	0.48	0.37, 0.59	<0.001
Gyroscope y-axis	0.41	0.32, 0.50	0.20	0.04, 0.36	0.54	0.45, 0.64	0.28	0.19, 0.37	0.51	0.41, 0.61	<0.001
Gyroscope z-axis	0.38	0.28, 0.48	0.19	0.04, 0.34	0.48	0.38, 0.58	0.25	0.15, 0.35	0.47	0.36, 0.57	<0.001

#### Standing.

Differentiation of standing behavior from other behaviors required reliance on readings from the x- and z-axes of the accelerometer, as well as the x-, y-, and z-axes of the magnetometer (Table 1). Werner et al. (2018) also found that accelerometer-based detection of standing behavior was possible using the RumiWatch System sensor platform. Similar results were found by Poulopoulou et al. (2019) evaluating the ability of a three triaxial accelerometer on predicting behavioral activities in grazing beef cattle. Although these studies all show that wearable technologies have promise as a means to classify standing behavior, the concordance correlation coefficient (CCC) to determine the accuracy between readings from the sensor and direct observations of existing evaluations is 0.88–0.97 (Werner et al., 2018; Poulopoulou et al., 2019). Leveraging this more flexible research platform for sensor evaluation may enable the development of algorithms to more reliably classify standing behavior.

#### Lying.

To differentiate confidently from laying patterns and other behaviors, readings from the x- and z-axes of the magnetometer (Table 1) were good candidates for classification. Sakai et al. (2019), evaluating the ability of a nine-axis multisensor equipped with a triaxial accelerometer, a triaxial gyroscope, and a triaxial magnetometer on classification behavior activities in goats, could classify laying behavior with 84% of accuracy. Similar results were found by Poulopoulou et al. (2019) using accelerometer data to classify behavior activities in grazing cattle, with 92% accuracy for laying patterns. Robert et al. (2009) evaluating the accelerometer attached to the hind leg of cattle predicted lying with 98% accuracy. Some studies have reported misclassification between standing and laying and grazing and laying (Marais et al., 2014; Barwick et al., 2018), which suggest improvements on algorithms on distinguishing resting behaviors are an important research objective.

#### Eating.

Differentiation between eating behavior from other behavior required reliance on readings from the x-, y-, and z-axes of the magnetometer (Table 1). Pereira et al. (2018) validating an ear tag accelerometer sensor on detecting grazing dairy cattle behavior, detected eating events with 88% accuracy. Similar results were reported in the study of Ruuska et al. (2018) evaluating automated systems for measuring feed behavior in dairy cattle; the sensor accuracy for feeding behavior was 81%. Eating behavior of direct observations and accelerometer sensor data were highly correlated (CCC = 0.82) in the study of Borchers et al. (2016). Magnetometers can be used with accelerometers to increase the metrics to quantify animal behavior because magnetometers are not sensitive to gravitational or dynamic acceleration (Noda et al., 2014). Guo et al. (2018) also identified that inertial measurement unit sensor equipped with a three-axis accelerometer, three-axis magnetometer, and three-axis gyroscope was able to differentiate among sheep grazing behaviors, where grazing was defined as the act of biting, chewing, or picking grass with the mouth while the head was down. In our study, eating patterns could not be differentiated based on accelerometer data as cited in the previous studies in cattle; however, the magnetometer data appeared sufficient for classification. The success in classifying eating behaviors in our study could be associated with different accelerometer patterns between sheep and cattle and the movements associated with biting and chewing. The sensor can be placed on different locations in the animal, which can influence the behavior classification and the data readings from the sensors and might have influenced the results of the present study.

#### Rumination.

The readings of x- and y-axes of the accelerometer, as well as the magnetometer x-axis, were required to differentiate rumination from other behaviors (Table 1). Borchers et al. (2016) evaluated the accuracy of various commercial sensors on detecting rumination in dairy cattle and reported a CCC = 0.59 with the CowManager SensOor system and a CCC = 0.96 with the Smartbow system. Pereira et al. (2018) evaluating an accelerometer-based sensor CowManager SensOor reported a CCC = 0.71 for rumination detection. The same system used by Bikker et al. (2014) with cattle had a high CCC = 0.93 between direct observations of rumination times and rumination estimated by the accelerometer. In a study validating the accuracy of the RumiWatch on differentiating behaviors in dairy cattle, Ruuska et al. (2018) found rumination behavior to be predicted with an accuracy of 89%. Giovanetti et al. (2017) tested the ability of a triaxial accelerometer with simultaneous video recordings to classify grazing, ruminating, and resting behaviors of dairy sheep in grazing conditions and found that the system could distinguish rumination with an accuracy of 95% and precision of 89%. A similar system was used by McLennan et al. (2015) to differentiate among specific activities performed by sheep. They reported that the accelerometer collar was only able to distinguish among active (walking, grazing, standing, and ruminating) and inactive behaviors (lying, lying, and ruminating). Automatic detection of rumination can be used to identify rumination disorders in ruminants. More reliable data to identify this activity can contribute to improvements in commercially available systems.

#### Drinking.

Differentiation of drinking behavior from other behaviors required reliance on readings from the x-, y-, and z-axes from the magnetometer (Table 1). Drinking behaviors were detected with a precision of 9% and an accuracy of 78% using an accelerometer system as reported by Poulopoulou et al. (2019). Low precision of accelerometer sensors on time spent drinking determined behavior were also reported by Ruuska et al. (2018) with 5.6% and with an accuracy of 98%. The relationship between drinking time by accelerometer system and continuously recorded behavior by video cameras were also poor (*R*^2^ = 0.20 as reported; Ruuska et al. 2016). Recording drinking behaviors have been demonstrated to be a challenge among current monitoring technologies; the results of the present study might contribute to developing algorithms to distinguish drinking patterns from other similar behaviors.

## CONCLUSIONS

The motivation of our study was to provide a low-cost open-source sensor capable of differentiating among animal behaviors and providing continuous monitoring of behavioral activities under different conditions. Even though the sensor was tested in housed sheep, it should be valid and feasible for monitoring behavior activities of any livestock species in any housing condition where Bluetooth transmissions can be reliable. The sensor could classify among standing, lying, eating, ruminating, and drinking by integrating the measurements of the accelerometer, magnetometer, and gyroscope, suggesting that it may be a useful research tool to conduct work focused on enhancing the utility of wearable animal sensors.
